# *Klebsiella pneumoniae* ghosts as a novel adjuvant drive dendritic cell maturation and antigen-specific TCM expansion for IFN-γ-mediated immune protection

**DOI:** 10.1128/iai.00335-25

**Published:** 2025-10-31

**Authors:** Zhongtian Zhu, Junchi Xu, Jie Yang, Haixia Tian, Hongmei Jiao

**Affiliations:** 1 Medical College, Yangzhou University/Jiangsu Key Laboratory of Experimental & Translational Non-coding RNA Research 38043, Yangzhou, Jiangsu, China; 2 The Fifth People’s Hospital of Suzhou (The Aﬃliated Infectious Disease Hospital of Soochow University), Suzhou, China; 3 Jiangsu Key Laboratory of Zoonosis/Jiangsu Co-Innovation Center for Prevention and Control of Important Animal Infectious Diseases and Zoonoses, Yangzhou, China; 4 Joint International Research Laboratory of Agriculture and Agri-Product Safety, Yangzhou, China; University of California San Diego School of Medicine, La Jolla, California, USA

**Keywords:** *K. pneumoniae* ghosts, dendritic cell maturation, antigen-specific response, vaccine adjuvant

## Abstract

*Klebsiella pneumoniae* (*K. pneumoniae*), a common nosocomial pathogen causing severe pulmonary infections, is often complicated by coinfections. Bacterial ghosts, which are empty bacterial cell envelopes, hold significant promise as vaccine adjuvants. This study aims to develop and evaluate a novel combination vaccine platform utilizing *K. pneumoniae* ghosts (KP ghosts) to explore their intrinsic immunogenic properties as both vaccine and natural adjuvants. In this study, we showed that KP ghosts enhanced maturation and activation of bone marrow-derived dendritic cells, increasing surface markers (CD40, CD80, CD86, and MHC II) and cytokine secretion (IL-1β, TNF-α, and IL-12p70). The KP ghost-based vaccine provided strong immune protection in mice, significantly improving survival rates and reducing bacterial loads in organs after bacterial challenge. Additionally, to assess the adjuvant potential of KP ghosts, C57BL/6 mice were co-immunized with KP ghosts and a model antigen, ovalbumin (OVA). In comparison to OVA alone, the combination of OVA and KP ghosts elicited higher levels of specific IgG antibodies. Furthermore, OVA combined with KP ghosts increased the expression of the early activation marker CD69 on T cells after *in vitro* antigen stimulation and raised the frequencies of central memory T cells (Tcm) and CD4^+^ IFN-γ^+^ T cells. In conclusion, KP ghosts are effective as both vaccine and adjuvant components, enhancing the innate immune response of dendritic cells and the antigen-specific response of T cells. These findings highlight KP ghosts as a dual-purpose vaccine/adjuvant platform for broader antibacterial vaccine development.

## INTRODUCTION

In international guidelines, bacteria resistant to more than three classes of antibiotics are defined as multidrug-resistant (MDR) (1). ESKAPE pathogens, including *Enterococcus faecium* (*E. faecium*), *Staphylococcus aureus* (*S. aureus*), *Klebsiella pneumoniae* (*K. pneumoniae*), *Acinetobacter baumannii* (*A. baumannii*), *Pseudomonas aeruginosa* (*P. aeruginosa*), and *Enterobacter* species, represent a group of MDR bacteria that pose a serious threat to human health (2). These pathogens have attracted widespread scientific attention globally, highlighting the urgent need for developing new vaccines against them.

A microbiological report from a tertiary hospital in China, covering 2012–2021, found that the most frequently isolated bacteria from respiratory specimens were *A. baumannii*, *K. pneumoniae*, and *P. aeruginosa* (3). These pathogens often cause single or mixed infections, leading to various respiratory diseases. Besides lung diseases, *K. pneumoniae* and other pathogens can also infect the lower biliary tract, urinary tract, surgical wounds, and bloodstream (4). Gram-negative bacteria accumulate antimicrobial resistance (AMR) genes through the transfer of plasmids and genetic elements (5). Data show that over 400 AMR genes have been identified in various *K. pneumoniae* genomes, significantly more than in other gram-negative bacteria (6). *K. pneumoniae* naturally produces enzymes that hydrolyze β-lactam antibiotics, including ampicillin and amoxicillin. The extended-spectrum β-lactamase resistance in *K. pneumoniae* has led to widespread use of carbapenem antibiotics, likely resulting in the emergence of carbapenemase-producing *K. pneumoniae* (7). The emergence of the colistin resistance gene mcr-1 in *K. pneumoniae* has nearly exhausted treatment options for MDR-KP (8). This issue is also affecting other gram-negative bacteria, with increasing resistance mechanisms observed in *A. baumannii* and *P. aeruginosa*.

Although several vaccine targets have been proposed in recent decades (9–12), no vaccine against *K. pneumoniae* or other *Enterobacteriaceae* has been licensed to date. *K. pneumoniae* bacterial capsule polysaccharide has been investigated as a vaccine target. TonB-dependent transporters involved in immune responses to these pathogens have been recently studied (10). Outer membrane proteins (OMPs) are candidate vaccine targets in both *A. baumannii* (13) and *A. hydrophila* (14). However, candidate vaccine strategies often fail in clinical settings due to safety concerns and insufficient immune protection. Therefore, safe and effective vaccine adjuvants are essential.

Bacterial ghosts (BGs) have been shown to be safe and effective as non-living vaccines against various infectious diseases (15–18). Controlled expression of the phiX174 lysis gene E in BG cells forms transmembrane tunnels, causing expulsion of cytoplasm containing the genome and plasmids (15, 18). Even highly sensitive and fragile structures, such as pili, remain intact after ghost formation (19). Without physically or chemically destroying the outer membrane, the membrane retains its antigenic structure similar to that of living bacteria. Additionally, bacterial cells provide an excellent platform for delivering immunogenic antigens (20), and bacterial membrane components, such as lipopolysaccharides, peptidoglycans, and lipid A, possess adjuvant properties (21). These studies demonstrate the adjuvant properties of BGs, establishing them as a promising new vaccine platform. Dendritic cells (DCs) are crucial antigen-presenting cells (APCs) that bridge the innate and adaptive immune responses. Upon activation and maturation, DCs undergo phenotypic and functional changes that enhance their ability to migrate to lymph nodes and activate downstream T lymphocytes. In this study, we developed a new vaccine strategy using *K. pneumoniae* ghosts (KP ghosts) and evaluated its dual immunogenic functions serving as a vaccine and an intrinsic adjuvant *in vitro* and *in vivo*. Utilizing murine immunization models, we assessed the protective efficacy of KP ghosts against infection and characterized their antigen presentation capabilities.

## MATERIALS AND METHODS

### Plasmid constructs, bacterial isolates, and animals

The plasmid pBBR1MCS-E, which carries the gene E from bacteriophage and the thermo-sensitive lambda pL/pR-cI857, was constructed following established procedures (22). Clinical isolate *K. pneumoniae* strain T952 was cultured at 37°C in Luria-Bertani (LB) agar or broth. Six-week-old C57BL/6 mice were obtained from the Comparative Medicine Centre of Yangzhou University, China.

### Production of KP ghosts

KP ghosts were produced as previously described (22). Briefly, *K. pneumoniae* strain T952 competent cells were transformed with pBBR1MCS-E using electroporation. Positive transformants were confirmed by restriction enzyme digestion and PCR. For KP ghosts production, single colonies were grown into LB broth (50 µg/mL kanamycin) at 28°C until the OD_600_ was approximately 0.5, then induced at 42°C. Subsequently, KP ghosts were harvested and lyophilized.

### Generation and stimulation of bone marrow-derived dendritic cells (BMDCs)

BMDCs were generated by culturing bone marrow cells harvested from C57BL/6 mice as previously described (22, 23). The purity of BMDCs was approximately 85% based on flow cytometric analyses. The harvested BMDCs were seeded into 24-well plates and treated with KP ghosts (5 × 10^5^ CFU), KP ghosts (5 × 10^6^ CFU), KP ghosts (5 × 10^7^ CFU), or LPS (100 ng/mL) for 48 h at 37°C. Expression of surface molecules on BMDCs was determined by incubating BMDCs with FITC-CD86, PE-CD80, PE-CD40, FITC-MHC-II, PerCP-Cy5.5 CD69, and PE-CCR7 antibodies (all from eBioscience, USA) for 30 min at 4°C. The cell samples were harvested, washed, and analyzed by flow cytometry (FCM) using FlowJo software (BD Biosciences, USA).

### Endocytosis assay

The BMDCs were incubated with 1 mg/mL FITC-Dextran at 37°C for 30 min as previously described (24). Subsequently, BMDCs were washed two times with PBS and analyzed using FCM. Additionally, a control at 4°C was conducted to rule out adhesion effects.

### Allogeneic mixed lymphocyte reaction (MLR) assay

Male BALB/c mice, aged 6 weeks, were obtained from the Animal Research Center at Yangzhou University in Jiangsu, China. Responder T cells were purified from splenic lymphocytes using a CD4^+^ T-cell isolation kit (Miltenyi Biotec, Germany) and labeled with CFSE (Thermo Fisher Scientific, USA) following the manufacturer’s protocol. Purified T cells were co-cultured with BMDCs at a 1:1 ratio in complete RPMI-1640 medium supplemented with 10% fetal bovine serum (FBS) under 5% CO_2_ at 37°C for 5 days and analyzed using FCM.

### Cell viability assay

KP ghosts cytotoxicity assay was conducted in BMDCs using the CCK-8 kit (Jiancheng Biotech, Nanjing, China) following the manufacturer’s protocol. Briefly, the cells were cultured in 96-well plates and stimulated with KP ghosts at cell-to-ghost ratios of 1:1, 1:10, 1:100, and 1:200. Non-stimulated cells served as the negative control. After 48 h of incubation at 37°C, 10 µL of CCK-8 was added per well, followed by incubation for 1 h. Absorbance at 450 nm was measured, and results were compared relative to the control group.

### Cytokine assay

BMDCs were cultured *in vitro* with KP ghosts or LPS for 24 h, followed by quantification of TNF-α, IL-1β, and IL-12p70 levels in the culture supernatants using enzyme-linked immunosorbent assay (ELISA) kits (Dakewe Biotech Co., Ltd., China) according to the manufacturer’s instructions.

### Bacterial challenge in immunized mice

Six-week-old C57BL/6 mice were divided into three groups, each of which included 15 mice. The mice were respectively immunized with PBS, KP ghosts (1 × 10^7^ CFU/animal), and KP formalin (1 × 10^7^ CFU/animal) with an initial vaccination at week 0, followed by boosts at weeks 2 and 4. In the challenge experiment, immunized mice were infected with *K. pneumoniae* T952 at a dose of 1 × 10^8^ CFU/animal at week 6. Body weight changes and survival rates were monitored for 12 days post-infection in each group of 10 mice. For the organ bacterial load experiment, immunized mice were infected with *K. pneumoniae* T952 at a dose of 2 × 10^7^ CFU/animal in week 6. At 48 h post-infection, mice were euthanized, and lungs and spleens were collected. The organs were homogenized, filtered, and resulting homogenates were serially diluted before plating on LB agar plates. Plates were incubated at 37°C for 12 h for bacterial colony enumeration.

### Immunization with ovalbumin (OVA) antigen and KP ghosts adjuvants in mice

Six-week-old C57BL/6 mice were randomly divided into five groups: A, B, C, D, and E. Group A mice were immunized subcutaneously with sterile PBS as a control. Group B mice were immunized subcutaneously with 1 × 10^7^ CFU of KP ghosts. Group C mice were immunized subcutaneously with 50 µg of OVA antigen (Sigma). Group D mice were immunized subcutaneously with 50 µg of OVA antigen plus 1 × 10^7^ CFU of KP ghosts. Group E mice were immunized subcutaneously with 50 µg of OVA antigen and aluminum hydroxide (alum) adjuvant (Biodragon, China). All mice received three immunizations at 2-week intervals. Two weeks after the final immunization, serum and spleen samples were collected for immunological analysis.

### ELISA

ELISA was performed to determine antibody levels as previously described (25), with minor modifications. Briefly, 96-well ELISA plates were coated overnight at 4°C with 100 µL of OVA (5 µg/mL) in carbonate-bicarbonate buffer (pH 9.6). After washing with PBST (PBS containing 0.05% Tween), the plates were blocked with PBS containing 10% FBS for 4 h at 37°C. After washing, serial dilutions of serum samples were added to the wells and incubated for 2 h at 37°C. The plates were then washed, and HRP-conjugated goat anti-mouse IgG antibody (1:8,000, Abcam) was added and incubated for 1 h at 37°C. After another wash, tetramethylbenzidine (Solarbio, Beijing, China) was added to each well. Finally, the reaction was stopped by adding 2 M H_2_SO_4_, and the OD_450_ values were measured using an ELISA plate reader. Antibody titers were reported as the logarithm of the reciprocal of the highest serum dilution with an OD value equal to or greater than 2.1 times the negative control.

### FCM analysis of memory T cells

Single-cell suspensions from spleens were prepared as described previously. The spleen cells were seeded into 24-well plates and treated with OVA (30 µg/mL) for 48 h. After antigen stimulation, the cells were incubated with APC-Cy7-conjugated anti-CD3, PE-conjugated anti-CD4, APC-conjugated anti-CD8α, FITC-conjugated anti-CD44, and PE-Cyanine7-conjugated anti-CD62L antibodies at 4°C for 30 min to analyze T-cell subpopulations. T-cell activation was assessed by incubating the cell suspensions with FITC-conjugated anti-CD3, PE-conjugated anti-CD4, APC-conjugated anti-CD8, and PerCP-Cy5.5-conjugated anti-CD69 antibodies (all from eBioscience) at 4°C for 30 min. After washing, the cells were analyzed using a flow cytometer and FlowJo software (BD Biosciences).

### Intracellular cytokine staining

Intracellular cytokine staining was conducted using the Cytofix/Cytoperm kit (BD Biosciences) according to the manufacturer’s protocol. Briefly, splenocytes were stimulated with OVA antigen for 48 h. Brefeldin A (BD Biosciences) was added during the last 5 h of stimulation to inhibit protein transport. The splenocytes were then incubated with FITC-conjugated anti-CD3 and APC-conjugated anti-CD4 antibodies at 4°C. Subsequently, the cells were stained with PE-conjugated anti-IFN-γ or PE-conjugated anti-IL-4 antibodies (all from eBioscience) to detect intracellular cytokines. Samples were analyzed using a flow cytometer and FlowJo software (BD Biosciences).

### Statistical analysis

Statistical analyses were conducted using GraphPad Prism 7.04 (GraphPad Software, Inc., La Jolla, CA, USA). One-way analysis of variance (ANOVA) with Tukey’s post hoc test was used to compare multiple groups. Unpaired two-sided Student’s *t*-test was used to compare two groups. *P* < 0.05 was considered statistically significant, and *P* < 0.01 was deemed highly significant.

## RESULTS

### Enhanced phenotypic maturation of BMDCs by KP ghosts

**Fig 1:**
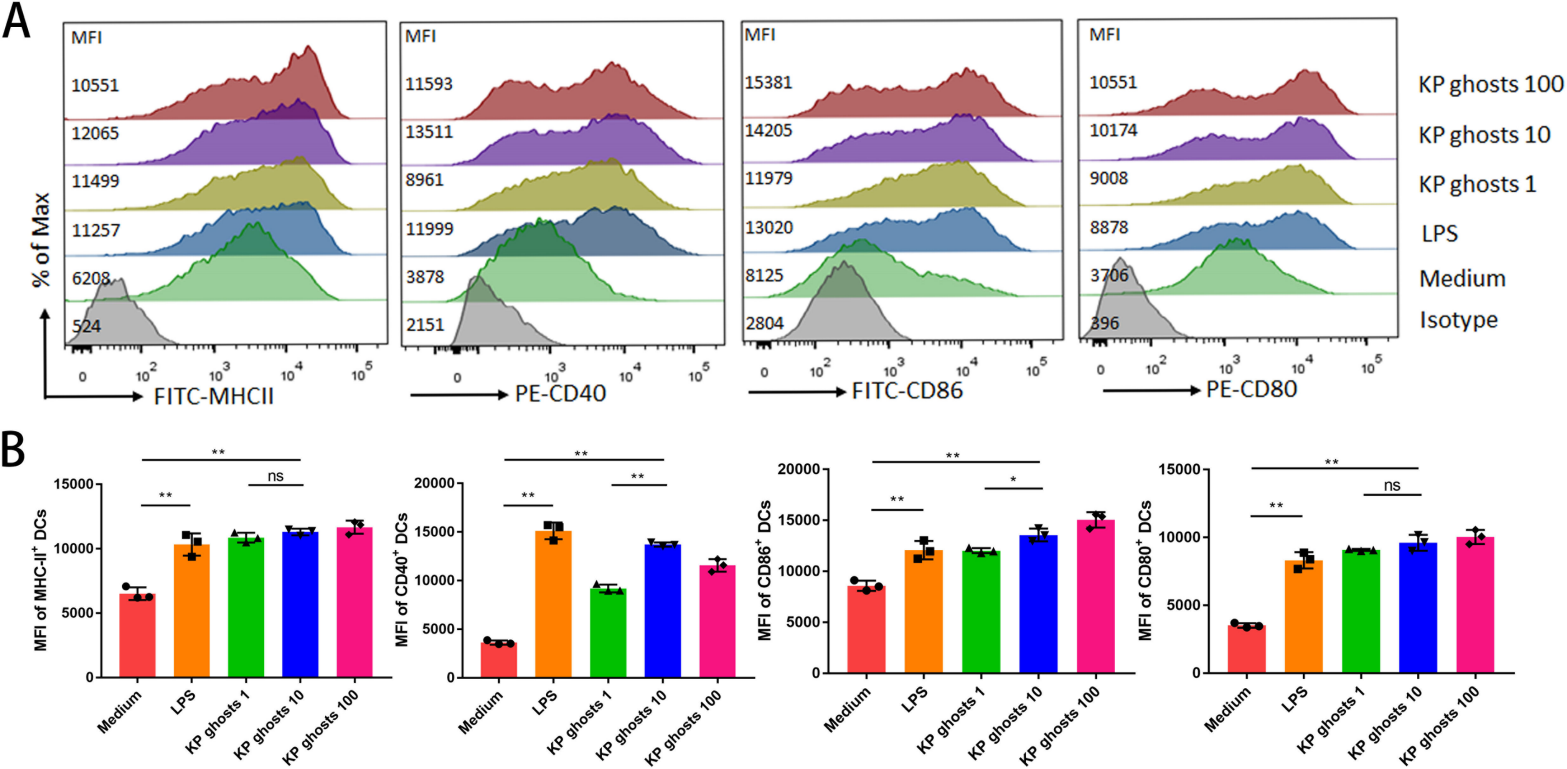
Expression of phenotypic markers on dendritic cells stimulated with KP ghosts *in vitro*. (**A, B**) Flow cytometry analysis of phenotypic markers MHC-II, CD40, CD80, and CD86 on BMDCs was performed after 48 h of stimulation with various proportions of KP ghosts or 100 ng/mL LPS. KP ghosts 1: KP ghosts to BMDCs ratio of 1:1; KP ghosts 10: ratio of 10:1; KP ghosts 100: ratio of 100:1. *n* = 3 per group. Data shown are from one representative experiment of three independent replicates. Values are expressed as mean ± standard deviation. Statistical significance was determined using one-way ANOVA. ns, *P* > 0.05; *P < 0.05; and **P < 0.01.

To produce KP ghosts, we constructed the lysis plasmid p-EBOX containing the E lysis cassette and transformed it into *K. pneumoniae* strain T952. The temperature was increased to 42°C to induce the KP ghosts production. DC phenotypic maturation is characterized by the expression of CD80, CD86, CD40, and MHC-II (26). BMDCs were incubated with either LPS or varying doses of KP ghosts, and the expression of phenotypic markers was assessed using FACS. Treatment with KP ghosts significantly upregulated surface expression of maturation markers (CD80, CD86, CD40, and MHC-II) on BMDCs compared to untreated controls (Fig. 1A and B). Notably, the upregulatory effects were dose-related, with the 10:1 KP ghosts-to-BMDC ratio inducing the most potent expression for CD40 and a significantly greater expression of CD86 than the 1:1 ratio. However, the maximal response for each marker was achieved at different doses, indicating a complex dose-response relationship rather than a simple linear dependency. These findings demonstrate that KP ghosts effectively promote phenotypic maturation of BMDCs.

### Enhancement of BMDCs functionality by KP ghosts

**Fig 2:**
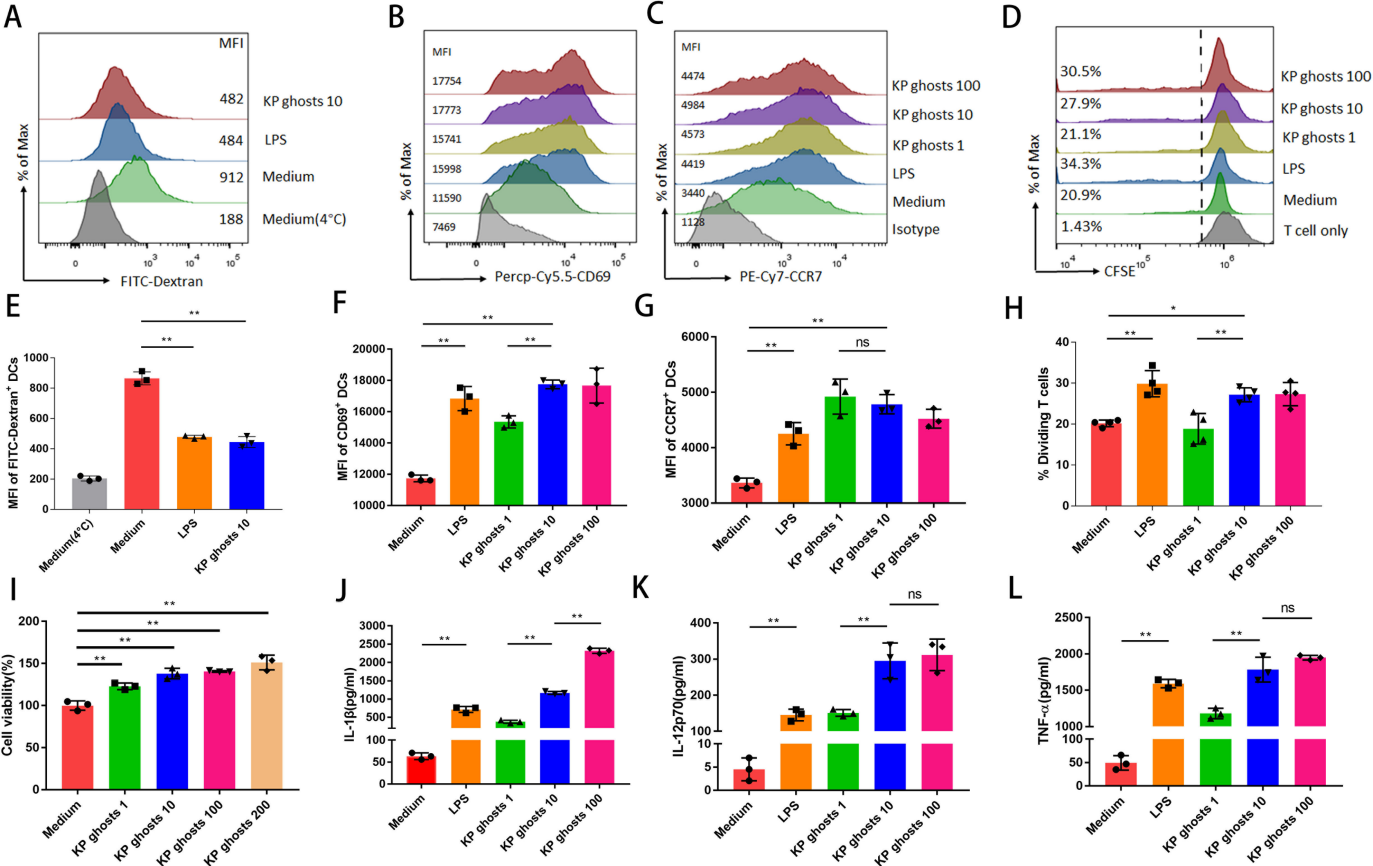
BMDCs were stimulated with various proportions of KP ghosts or 100 ng/mL LPS to assess endocytosis, activation and migration phenotypes, and cytokine responses. Additionally, the proliferative effects of stimulated dendritic cells on allogenic T cells were evaluated. (**A, E**) After 48 h of stimulation with KP ghosts or LPS, treated BMDCs were incubated with 1 mg/mL FITC-Dextran at 37°C for 30 min. Endocytosis was assessed by flow cytometry, with LPS serving as a positive control. (**B, F**) CD69 expression was measured by flow cytometry in treated BMDCs after 48 h of stimulation with KP ghosts or LPS. (**C, G**) CCR7 expression was measured by flow cytometry in treated BMDCs after 48 h of stimulation with KP ghosts or LPS. (**D, H**) After 48 h of KP ghosts or LPS stimulation, treated BMDCs were co-cultured with CFSE-labeled naive CD4^+^ allogenic T cells at a 1:1 ratio (1 × 10^5^ T cells per well). Cell proliferation was assessed by flow cytometry after 5 days. (**I**) BMDCs were stimulated with KP ghosts at ratios of 1:1, 1:10, 1:100, and 1:200 for 48 h. Cell viability was then measured using the CCK-8 assay. (**J, K, L**) After 48 h, the BMDC supernatant was collected, and cytokine levels (IL-1β, IL-12p70, and TNF-α) were measured using an ELISA kit. KP ghosts 1: KP ghosts to BMDCs ratio of 1:1; KP ghosts 10: ratio of 10:1; KP ghosts 100: ratio of 100:1. *n* = 3–4 per group, as indicated. Data shown are from one representative experiment of three independent replicates. Values are expressed as mean ± standard deviation. Statistical significance was determined using one-way ANOVA. ns, *P* > 0.05; *P < 0.05; and **P < 0.01.

The toxicity of KP ghosts was assessed in murine DCs. Cells were treated with KP ghosts, and cell viability/metabolic activity was evaluated using the CCK-8 assay. Results showed that KP ghost treatment did not reduce cell viability; instead, a modest increase in metabolic activity was observed after 48 h of incubation (Fig. 2I). Inflammatory stimuli induce maturation in BMDCs, characterized by downregulation of endocytosis (27). The fluorescent marker dextran was used to assess whether KP ghosts modulated BMDC endocytosis. As depicted in Fig. 2A and E, KP ghosts significantly reduced BMDC endocytosis compared to the untreated control, with LPS as a positive control.

After 48 h of incubation with KP ghosts, BMDCs showed enhanced CD69 expression, indicating activation (Fig. 2B and F). Inflammatory mediators stimulate BMDC maturation and migration from non-lymphoid to lymphoid organs to initiate T-cell-mediated immune responses. This migration is closely associated with BMDC expression of CCR7 (28). Expression levels of CCR7 in BMDCs were analyzed by FCM to assess whether KP ghosts modulated BMDC migration. As shown in Fig. 2C and G, CCR7 expression significantly increased in all KP ghosts-treated groups, with no significant differences between groups. Furthermore, levels of secreted IL-1β, IL-12p70, and TNF-α were significantly higher in KP ghosts-treated BMDCs compared to untreated cells (Fig. 2J, K and L). Overall, KP ghosts enhanced BMDC activation, migration capability, and pro-inflammatory cytokine response.

DCs are potent stimulators of allogeneic T-cell proliferation in the MLR (29). To assess the effects of KP ghosts on MLR stimulation ability, BMDCs were collected and co-cultured with allogeneic CD4^+^ T cells. As depicted in Fig. 2D and H, KP ghosts-induced BMDCs effectively stimulated proliferative responses compared to untreated BMDCs. A ratio of 1:10 BMDCs to KP ghosts stimulates MLR more effectively than a 1:1 ratio. These results suggest that KP ghosts significantly enhance the allostimulatory capacity of BMDCs.

### Protective efficacy of KP ghosts against *K. pneumoniae* challenge

**Fig 3:**
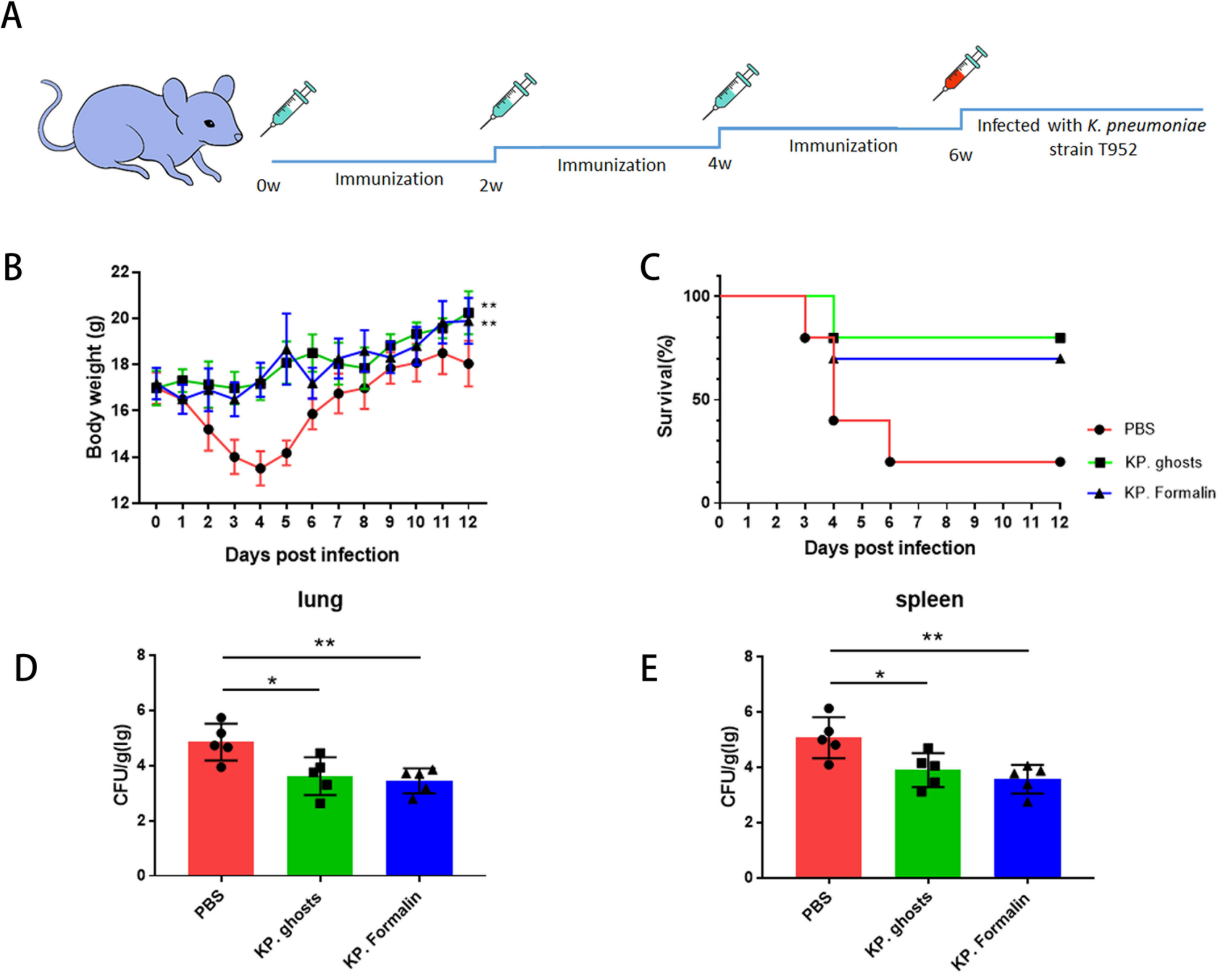
KP ghosts offer enhanced specific immune protection compared to the inactivated *K. pneumoniae* vaccine. (**A**) Establishment of the mouse infection model. Six-week-old C57BL/6 mice received three intraperitoneal booster doses of identical vaccine components at 2-week intervals over 6 weeks. At 6 weeks post-immunization, mice were intraperitoneally challenged with *K. pneumoniae* to establish the peritoneal infection model. (**B, C**) Two weeks after the third immunization, mice were intraperitoneally challenged with a lethal dose of *K. pneumoniae* strain T952. (**B**) Body weight changes of surviving mice over 12 days post-infection for each group. (**C**) Survival rates and survival curves. *n* = 10 mice per group. (**D, E**) Two weeks after the third immunization, mice were intraperitoneally challenged with a sub-lethal dose of *K. pneumoniae* strain T952. (**D**) Bacterial colony counts from lung homogenates prepared 48 h post-infection, plated on LB agar. (**E**) Bacterial colony counts from spleen homogenates, also prepared 48 h post-infection. Data were presented as the mean ± standard deviation and are representative of three independent experiments (*n* = 5 mice per group). Statistical significance was determined using one-way ANOVA. ns, *P* > 0.05; *P < 0.05; and **P < 0.01.

At weeks 0, 2, and 4, mice were immunized with PBS, KP ghosts (1 × 10⁷ CFU/animal), or KP formalin (1 × 10⁷ CFU/animal), followed by a 2-week resting period. At week 6 post-initial immunization, all groups were challenged with a lethal dose of *K. pneumoniae* strain T952 via intraperitoneal injection (Fig. 3A). Mice in the control group exhibited severe symptoms, including rapid weight loss, and had an 80% mortality rate. In contrast, the KP ghosts group showed only a few cases of illness, with no significant weight loss, and had a 20% mortality rate. The KP formalin group had a mortality rate of 30% (Fig. 3B and C). To further assess the protective efficacy of KP ghost vaccination, mice were challenged with a sub-lethal dose of *K. pneumoniae*, and bacterial burdens in the lung and spleen were determined. Our results showed that organ bacterial loads in the immunized groups were significantly lower than those in the control group (Fig. 3D and E).

### Enhanced induction of OVA-specific IgG antibodies by KP ghosts

**Fig 4:**
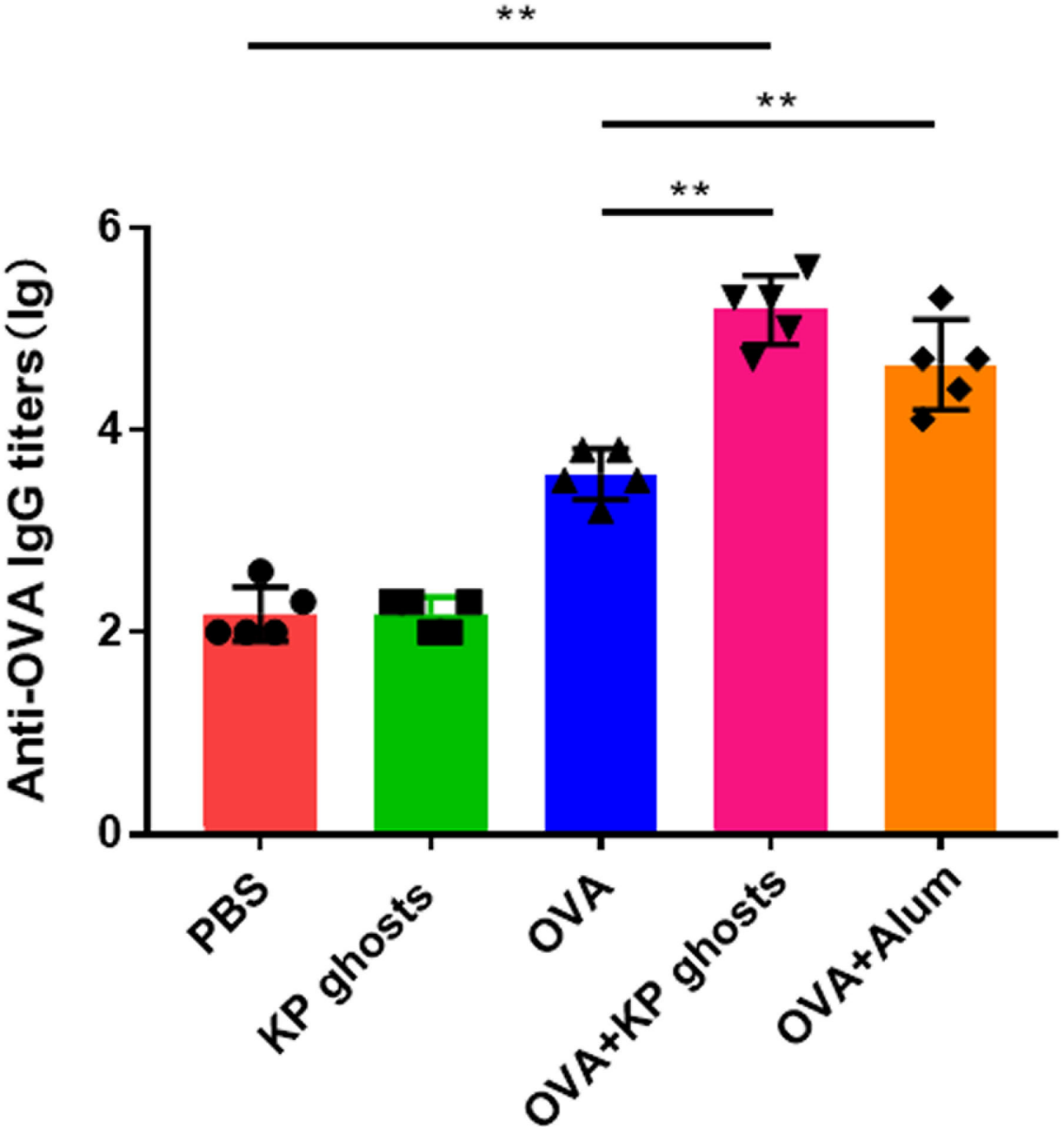
KP ghosts induced higher IgG antibody levels compared to the OVA antigen alone. Mice were divided into five groups and immunized with PBS, KP ghosts, OVA, KP ghosts + OVA, or Alum + OVA. All groups received three immunizations at 2-week intervals. Two weeks after the final immunization, serum samples were collected and analyzed by ELISA to determine OVA-specific IgG antibody levels. Data were presented as the mean ± standard deviation and are representative of three independent experiments (*n* = 5 mice per group). Statistical significance was determined using one-way ANOVA. ns, *P* > 0.05; *P < 0.05; and **P < 0.01.

To investigate the role of KP ghosts in inducing OVA-specific humoral immune responses, we developed a mouse model using OVA as the target antigen. Alum served as the positive control, and PBS served as the negative control. OVA-specific IgG antibodies in the peripheral blood serum of the immunized mice were measured by ELISA. Mice immunized with KP ghosts + OVA or Alum + OVA exhibited significantly higher IgG antibody responses compared to those receiving only OVA. KP ghosts demonstrated adjuvant-like functions similar to alum, leading to increased specific antibody levels and humoral immune responses compared to OVA alone. No antibody response was observed in the PBS group (Fig. 4).

### Effects of KP ghosts on T-cell populations and activation

**Fig 5:**
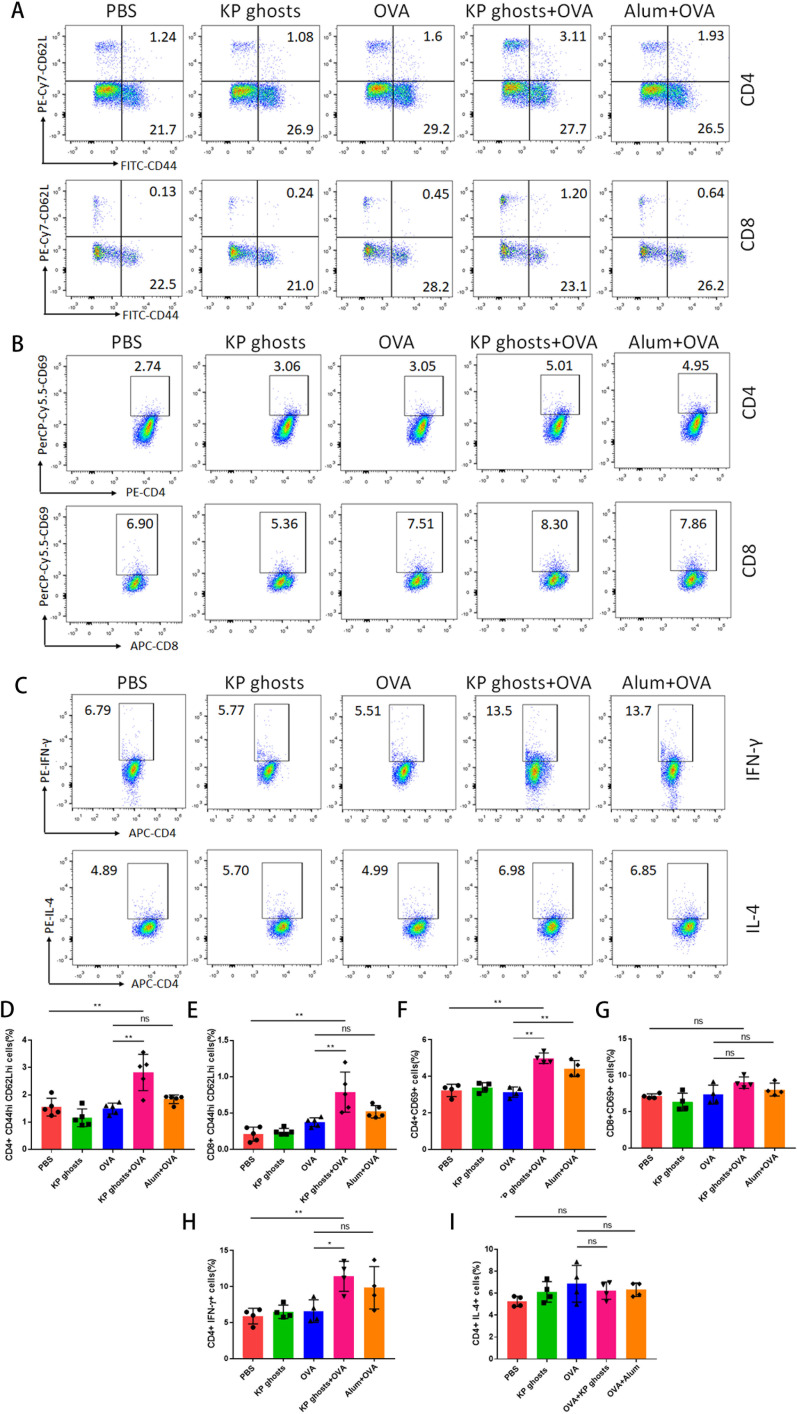
KP ghosts markedly enhance T cell-specific antigen recognition, promote the proliferation or differentiation of antigen-specific Tcm, and increase CD4^+^ T-cell activation and IFN-γ release. (**A, D, E**) Two weeks after the final immunization, splenocytes were collected and stimulated with OVA for 48 h. Flow cytometry was employed to analyze antigen-specific T-cell populations. (**A**) Representative FACS plots of CD44^hi^ CD62L^hi^ Tcm in immunized mice. (**D and E**) Percentages of Tcm (CD44^hi^ CD62L^hi^) among CD4^+^ and CD8^+^ T cells, respectively. (**B, F, G**) Splenocytes were isolated and stimulated as in panel **A** to assess early T-cell activation. (**B**) Representative flow cytometry plots of CD69 expression on CD4^+^ and CD8^+^ T cells. (**F, G**) Quantification of the percentage of CD69^+^ cells among CD4^+^ (**F**) and CD8^+^ (**G**) T cells. (**C, H, I**) Splenocytes were isolated and stimulated as in panel **A** to analyze antigen-specific cytokine production. (**C**) Representative flow cytometry plots of IFN-γ and IL-4 production by CD4^+^ T cells. (**H, I**) Quantification of the percentage of IFN-γ^+^ (**H**) and IL-4^+^ (**I**) cells among CD4^+^ T cells. Data were presented as the mean ± standard deviation and are representative of three independent experiments (*n* = 4–5 mice per group). Statistical significance was determined using one-way ANOVA. ns, *P* > 0.05; *P < 0.05; and **P < 0.01.

To further evaluate the effect of KP ghosts on T-cell populations, we conducted flow cytometric analysis to determine the proportions of central memory T cells (CD44^hi^ CD62L^hi^) and effector memory T cells (CD44^hi^ CD62L^lo^) in the spleens of immunized mice following antigen stimulation. After *in vitro* culture of splenocytes and stimulation with OVA for 48 h, KP ghosts + OVA-immunized mice showed a significant increase in the percentage of CD4^+^ CD44^hi^ CD62L^hi^ and CD8^+^ CD44^hi^ CD62L^hi^ T cells compared to the OVA-only group (Fig. 5A, D and E). This indicates an expansion of the central memory T-cell population in response to OVA-specific antigen stimulation.

To evaluate T-cell activation, we measured the expression of the early activation marker CD69 using flow cytometry. Figure 5B, F and G show that the frequency of CD4^+^ CD69^+^ T cells was significantly higher in the KP ghosts + OVA group compared to the OVA-only group, while no significant difference was observed in the frequency of CD8^+^ CD69^+^ T cells. This suggests a marked activation of CD4^+^ T cells in the KP ghosts + OVA group following stimulation with OVA-specific antigen.

Next, we performed intracellular cytokine staining to measure IFN-γ and IL-4 production in CD4^+^ T cells from the spleen. Figure 5C, H and I show that the percentage of CD4^+^ IFN-γ^+^ cells in the KP ghosts + OVA group was significantly higher than in the OVA-only group, while there was no significant difference in the percentage of CD4^+^ IL-4^+^ cells between the groups. These results suggest that KP ghosts enhance IFN-γ production by CD4^+^ T cells in response to OVA-specific antigen stimulation, without affecting IL-4 production.

## DISCUSSION

*K. pneumoniae* is a prevalent nosocomial pathogen responsible for pneumonia, urinary tract infections, and surgical wound infections. It is highly resilient and resistant to antibiotics. The accumulation of AMR over time has worsened resistance, highlighting the need for effective vaccine strategies to combat *K. pneumoniae*.

BGs are a novel type of inactivated vaccine, characterized by their surface transmembrane channels and leakage of nucleic acids and proteins. Compared to traditional vaccines, BGs differ significantly in proteomics and antibody profiles, enriched in immune proteins such as OMPs, heat shock proteins, and SodC. The immune response induced by BGs also differs markedly (30). BGs preserve sensitive and fragile structures, such as pili, during their gentle preparation process, unlike traditional vaccines, which often cause physical or chemical damage (31). Scanning electron microscopy reveals that BGs retain their complete cell structures and exhibit transmembrane channels, distinguishing them from the original strains (32).

Toll-like receptors (TLRs) are critical in developing new vaccines by initiating immune responses against pathogens (33). BGs, which have surface structures resembling live bacteria and contain TLR agonists, are effectively recognized by APCs. BGs can function as potent vaccines or adjuvants, inducing robust immune responses. Studies show that DCs mature with increased expression of surface markers such as CD40, MHC-II, CD80, and CD86 (34), reduced endocytosis, and increased cytokine release (35, 36). After maturation, DCs also exhibit increased CCR7 expression (37), which enhances their migration to lymph nodes for antigen presentation. KP ghosts stimulate BMDCs to upregulate surface markers and migration molecules and enhance cytokine secretion and T-cell proliferation. At a BMDCs to KP ghosts ratio of 1:10, BMDC maturation is maximized, with no significant difference compared to the 1:100 ratio. Excessive antigen levels may cause adverse vaccine reactions (38). Therefore, the 1:10 ratio of KP ghosts is identified as the optimal dose in this study. These findings suggest that KP ghosts effectively promote BMDC maturation, exhibit strong immunogenicity, and are a valuable reference for candidate vaccines or adjuvants.

BGs have been proven to safely and effectively prevent a range of infectious diseases (39, 40). This study shows that KP ghosts provide strong immune protection in mice, achieving an 80% survival rate under lethal bacterial challenge, compared to 20% in the control group. Bacterial load is a key parameter for assessing murine infection models (41). Both KP ghosts and inactivated vaccines reduce bacterial loads in the lungs and spleen of mice, providing significant immune protection.

Naive T cells are activated and differentiate into distinct phenotypes—central memory T cells (Tcm: CD44^hi^ CD62L^hi^) and effector memory T cells (Tem: CD44^hi^ CD62L^lo^)—upon initial antigen or infection stimulation (42, 43). Tem cells migrate to inflamed peripheral tissues to mediate protective memory and provide immediate defense, whereas Tcm cells return to secondary lymphoid organs to mediate secondary responses and offer long-term protection (44, 45). Two weeks after immunization, without further antigen stimulation, mouse immune cells stabilize, and no significant differences are observed among splenic memory T-cell subsets. Re-stimulation with OVA antigen *in vitro* induces proliferation and activation of antigen-specific memory T cells. This study found that the KP ghosts + OVA group had a significantly higher frequency of CD4^+^ and CD8^+^ Tcm subsets compared to the OVA group. The expansion of central memory T cells correlates with improved local immune protection (46). Notably, we observed the emergence of a CD44^lo^ CD62L^lo^ population after 48 h of *in vitro* culture. This atypical subset is rarely present in freshly isolated splenocytes, which displayed the expected distribution of naive (CD44^lo^ CD62L^hi^), effector memory (CD44^hi^ CD62L^lo^), and central memory (CD44^hi^ CD62L^hi^) T cells. While the precise biological significance of this population remains unclear, it may reflect culture-induced stress, altered activation dynamics, or functional exhaustion and warrants further investigation. Importantly, within the same baseline, our data robustly demonstrate that combined immunization significantly upregulated the CD44^hi^ CD62L^hi^ Tcm subset in mouse splenocytes following antigen stimulation.

After OVA stimulation, CD69 expression in CD4^+^ T cells was significantly higher in the KP ghosts + OVA group compared to the OVA group, indicating antigen-specific activation of CD4^+^ T cells, with no similar increase in CD8^+^ T cells. Additionally, IFN-γ secretion in activated CD4^+^ T cells increased significantly, with no corresponding rise in IL-4. CD4^+^ T helper (Th) cells comprise subsets including Th1, Th2, and Th17. Th1 cells release IFN-γ and express T-BET, which is involved in type I immune responses (47, 48). In this study, KP ghosts as an adjuvant increased IFN-γ secretion in OVA-specific CD4^+^ T cells, skewing the response toward a Th1-type reaction. This suggests that using KP ghosts as an adjuvant may enhance vaccine efficacy in clearing intracellular bacteria and viruses, offering potential clinical benefits. In conclusion, our research shows that KP ghosts as an adjuvant effectively enhance antigen-specific Tcm expansion, activate cellular immunity, and induce CD4^+^ T-cell activation and IFN-γ release, thereby promoting adaptive memory immune responses against pathogens.

KP ghosts significantly enhance the immunogenicity of the OVA antigen when used as an adjuvant, boosting antibody levels and elevating CD4^+^ T-cell immune responses. However, the mechanisms underlying this enhancement remain unclear. These ghost particles contain substantial amounts of LPS, lipoproteins, peptidoglycans, and pili. These pathogen-associated molecular patterns (PAMPs) (49) are recognized by pattern recognition receptors on immune cells. The recognition of these PAMPs stimulates the production of various immune mediators and promotes the maturation of APCs. For instance, LPS activates TLR4 via MyD88-dependent or MyD88-independent pathways (50, 51), leading to DC maturation and the differentiation of T cells into Th1 cells (52). Additionally, ghosts can protect internal molecules from degradation and gradually release them at the cell membrane. This property may facilitate the targeted delivery of nucleic acids, proteins, and chemical drugs (53, 54). Furthermore, studies indicate that live or inactivated forms of bacteria, such as BCG and *A. baumannii*, can induce cross-protective effects against other pathogens, a phenomenon known as trained immunity (55, 56). Considering the role of KP ghosts in enhancing vaccine immunogenicity and their potential for cross-protective immunity, further investigation into their mechanisms as a vaccine formulation could clarify their functions and offer valuable insights for developing new vaccines. Understanding these mechanisms may refine strategies for developing vaccines that utilize ghost-based adjuvants.

In summary, our research highlights the significant role of the KP ghosts in enhancing the innate immune response of BMDCs and their effectiveness in preventing *K. pneumoniae* infections while alleviating associated symptoms. The evaluation of a combined vaccine strategy, utilizing the KP ghosts as an adjuvant alongside recombinant antigen OVA, demonstrated the ability to induce a strong T-cell adaptive immune response, improve antigen immunogenicity, and significantly boost OVA antibody levels. These findings offer valuable insights and strategies for the future development of vaccines targeting *K. pneumoniae* and other clinical pathogens.

## Data Availability

The data are available from the corresponding author on reasonable request.

## References

[1] Magiorakos A-P, Srinivasan A, Carey RB, Carmeli Y, Falagas ME, Giske CG, Harbarth S, Hindler JF, Kahlmeter G, Olsson-Liljequist B, Paterson DL, Rice LB, Stelling J, Struelens MJ, Vatopoulos A, Weber JT, Monnet DL. 2012. Multidrug-resistant, extensively drug-resistant and pandrug-resistant bacteria: an international expert proposal for interim standard definitions for acquired resistance. Clin Microbiol Infect 18:268–281. doi:10.1111/j.1469-0691.2011.03570.x 21793988

[2] Ma Y, Wang C, Li Y, Li J, Wan Q, Chen J, Tay FR, Niu L. 2020. Considerations and caveats in combating ESKAPE pathogens against nosocomial infections. Adv Sci (Weinh) 7:1901872. doi:10.1002/advs.201901872 31921562 PMC6947519

[3] Shi T, Xie L. 2023. Distribution and antimicrobial resistance analysis of gram-negative bacilli isolated from a tertiary hospital in Central China: a 10-year retrospective study from 2012 to 2021. Front Microbiol 14:1297528. doi:10.1038/s41422-020-00395-4 38111644 PMC10726009

[4] Paczosa MK, Mecsas J. 2016. Klebsiella pneumoniae: going on the offense with a strong defense. Microbiol Mol Biol Rev 80:629–661. doi:10.1128/MMBR.00078-15 27307579 PMC4981674

[5] Navon-Venezia S, Kondratyeva K, Carattoli A. 2017. Klebsiella pneumoniae: a major worldwide source and shuttle for antibiotic resistance. FEMS Microbiol Rev 41:252–275. doi:10.1093/femsre/fux013 28521338

[6] Wyres KL, Holt KE. 2018. Klebsiella pneumoniae as a key trafficker of drug resistance genes from environmental to clinically important bacteria. Curr Opin Microbiol 45:131–139. doi:10.1016/j.mib.2018.04.004 29723841

[7] Martin RM, Bachman MA. 2018. Colonization, infection, and the accessory genome of Klebsiella pneumoniae. Front Cell Infect Microbiol 8:4. doi:10.3389/fcimb.2018.00004 29404282 PMC5786545

[8] McGann P, Snesrud E, Maybank R, Corey B, Ong AC, Clifford R, Hinkle M, Whitman T, Lesho E, Schaecher KE. 2016. Escherichia coli harboring mcr-1 and bla_CTX-M_ on a novel IncF plasmid: first report of mcr-1 in the United States. Antimicrob Agents Chemother 60:4420–4421. doi:10.1128/AAC.01103-16 27230792 PMC4914657

[9] Choi M, Tennant SM, Simon R, Cross AS. 2019. Progress towards the development of Klebsiella vaccines. Expert Rev Vaccines 18:681–691. doi:10.1080/14760584.2019.1635460 31250679 PMC6656602

[10] Wang Y-Q, Bazin-Lee H, Evans JT, Casella CR, Mitchell TC. 2020. MPL adjuvant contains competitive antagonists of human TLR4. Front Immunol 11:577823. doi:10.3389/fimmu.2020.577823 33178204 PMC7596181

[11] Kawahara K. 2021 Variation, modification and engineering of lipid A in endotoxin of gram-negative bacteria. Int J Mol Sci 22:2281. doi:10.3390/ijms22052281 33668925 PMC7956469

[12] Yang N, Jin X, Zhu C, Gao F, Weng Z, Du X, Feng G. 2022. Subunit vaccines for Acinetobacter baumannii. Front Immunol 13:1088130. doi:10.3389/fimmu.2022.1088130 36713441 PMC9878323

[13] Tamehri M, Rasooli I, Pishgahi M, Jahangiri A, Ramezanalizadeh F, Banisaeed Langroodi SR. 2022. Combination of BauA and OmpA elicit immunoprotection against Acinetobacter baumannii in a murine sepsis model. Microb Pathog 173:105874. doi:10.1016/j.micpath.2022.105874 36356792

[14] Zhang M, Zhang T, He Y, Cui H, Li H, Xu Z, Wang X, Liu Y, Li H, Zhao X, Cheng H, Xu J, Chen X, Ding Z. 2023. Immunogenicity and protective efficacy of OmpA subunit vaccine against Aeromonas hydrophila infection in Megalobrama amblycephala: an effective alternative to the inactivated vaccine. Front Immunol 14:1133742. doi:10.3389/fimmu.2023.1133742 36969197 PMC10034085

[15] Kwon SR, Nam YK, Kim SK, Kim KH. 2006. Protection of tilapia (Oreochromis mosambicus) from edwardsiellosis by vaccination with Edwardsiella tarda ghosts. Fish Shellfish Immunol 20:621–626. doi:10.1016/j.fsi.2005.08.005 16226892

[16] Peng W, Si W, Yin L, Liu H, Yu S, Liu S, Wang C, Chang Y, Zhang Z, Hu S, Du Y. 2011. Salmonella enteritidis ghost vaccine induces effective protection against lethal challenge in specific-pathogen-free chicks. Immunobiology 216:558–565. doi:10.1016/j.imbio.2010.10.001 21247655

[17] Lubitz W, Witte A, Eko FO, Kamal M, Jechlinger W, Brand E, Marchart J, Haidinger W, Huter V, Felnerova D, Stralis-Alves N, Lechleitner S, Melzer H, Szostak MP, Resch S, Mader H, Kuen B, Mayr B, Mayrhofer P, Geretschläger R, Haslberger A, Hensel A. 1999. Extended recombinant bacterial ghost system. J Biotechnol 73:261–273. doi:10.1016/s0168-1656(99)00144-3 10486935

[18] Szostak MP, Hensel A, Eko FO, Klein R, Auer T, Mader H, Haslberger A, Bunka S, Wanner G, Lubitz W. 1996. Bacterial ghosts: non-living candidate vaccines. J Biotechnol 44:161–170. doi:10.1016/0168-1656(95)00123-9 8717400

[19] Eko FO, Witte A, Huter V, Kuen B, Fürst-Ladani S, Haslberger A, Katinger A, Hensel A, Szostak MP, Resch S, Mader H, Raza P, Brand E, Marchart J, Jechlinger W, Haidinger W, Lubitz W. 1999. New strategies for combination vaccines based on the extended recombinant bacterial ghost system. Vaccine (Auckl) 17:1643–1649. doi:10.1016/S0264-410X(98)00423-X 10194817

[20] Tabrizi CA, Walcher P, Mayr UB, Stiedl T, Binder M, McGrath J, Lubitz W. 2004. Bacterial ghosts – biological particles as delivery systems for antigens, nucleic acids and drugs. Curr Opin Biotechnol 15:530–537. doi:10.1016/j.copbio.2004.10.004 15560979

[21] Huter V, Hensel A, Brand E, Lubitz W. 2000. Improved protection against lung colonization by Actinobacillus pleuropneumoniae ghosts: characterization of a genetically inactivated vaccine. J Biotechnol 83:161–172. doi:10.1016/s0168-1656(00)00310-2 11000472

[22] Jiao H, Yang H, Zhao D, He L, Chen J, Li G. 2016. The enhanced immune responses induced by Salmonella enteritidis ghosts loaded with Neisseria gonorrhoeae porB against Salmonella in mice. FEMS Microbiol Lett 363:22. doi:10.1093/femsle/fnw239 27797865

[23] Ahmed M, Jiao H, Domingo-Gonzalez R, Das S, Griffiths KL, Rangel-Moreno J, Nagarajan UM, Khader SA. 2017. Rationalized design of a mucosal vaccine protects against Mycobacterium tuberculosis challenge in mice. J Leukoc Biol 101:1373–1381. doi:10.1189/jlb.4A0616-270R 28258153 PMC5433857

[24] Lutz MB, Aßmann CU, Girolomoni G, Ricciardi‐Castagnoli P. 1996. Different cytokines regulate antigen uptake and presentation of a precursor dendritic cell line. Eur J Immunol 26:586–594. doi:10.1002/eji.1830260313 8605925

[25] Lin L, Tan B, Pantapalangkoor P, Ho T, Hujer AM, Taracila MA, Bonomo RA, Spellberg B. 2013. Acinetobacter baumannii rOmpA vaccine dose alters immune polarization and immunodominant epitopes. Vaccine (Auckl) 31:313–318. doi:10.1016/j.vaccine.2012.11.008 PMC3557524 23153442

[26] Chen S, Miao X, Huangfu D, Zhao X, Zhang M, Qin T, Peng D, Liu X. 2021. H5N1 avian influenza virus without 80–84 amino acid deletion at the NS1 protein hijacks the innate immune system of dendritic cells for an enhanced mammalian pathogenicity. Transbound Emerg Dis 68:2401–2413. doi:10.1111/tbed.13904 33124785

[27] Platt CD, Ma JK, Chalouni C, Ebersold M, Bou-Reslan H, Carano RAD, Mellman I, Delamarre L. 2010. Mature dendritic cells use endocytic receptors to capture and present antigens. Proc Natl Acad Sci USA 107:4287–4292. doi:10.1073/pnas.0910609107 20142498 PMC2840134

[28] Yanagihara S, Komura E, Nagafune J, Watarai H, Yamaguchi Y. 1998. EBI1/CCR7 is a new member of dendritic cell chemokine receptor that is up-regulated upon maturation. J Immunol 161:3096–3102. doi:10.4049/jimmunol.161.6.3096 9743376

[29] Freudenthal PS, Steinman RM. 1990. The distinct surface of human blood dendritic cells, as observed after an improved isolation method. Proc Natl Acad Sci USA 87:7698–7702. doi:10.1073/pnas.87.19.7698 2145580 PMC54815

[30] He C-Y, Yang J-H, Ye Y-B, Zhao H-L, Liu M-Z, Yang Q-L, Liu B-S, He S, Chen Z-L. 2022. Proteomic and antibody profiles reveal antigenic composition and signatures of bacterial ghost vaccine of Brucella abortus A19. Front Immunol 13:874871. doi:10.3389/fimmu.2022.874871 35529865 PMC9074784

[31] Eko FO, Mayr UB, Attridge SR, Lubitz W. 2000. Characterization and immunogenicity of Vibrio cholerae ghosts expressing toxin-coregulated pili. J Biotechnol 83:115–123. doi:10.1016/S0168-1656(00)00315-1 11000467

[32] Hjelm A, Söderström B, Vikström D, Jong WSP, Luirink J, de Gier J-W. 2015. Autotransporter-based antigen display in bacterial ghosts. Appl Environ Microbiol 81:726–735. doi:10.1128/AEM.02733-14 25398861 PMC4277592

[33] Kaur A, Baldwin J, Brar D, Salunke DB, Petrovsky N. 2022. Toll-like receptor (TLR) agonists as a driving force behind next-generation vaccine adjuvants and cancer therapeutics. Curr Opin Chem Biol 70:102172. doi:10.1016/j.cbpa.2022.102172 35785601

[34] Levene RE, Shrestha SD, Gaglia MM. 2021. The influenza A virus host shutoff factor PA-X is rapidly turned over in a strain-specific manner. J Virol 95:e02312-20. doi:10.1128/JVI.02312-20 33504608 PMC8103685

[35] Schlitzer A, Zhang W, Song M, Ma X. 2018. Recent advances in understanding dendritic cell development, classification, and phenotype. F1000Res 7:1558. doi:10.12688/f1000research.14793.1 PMC6173131 30345015

[36] Fu C, Jiang A. 2018. Dendritic cells and CD8 T cell immunity in tumor microenvironment. Front Immunol 9:3059. doi:10.3389/fimmu.2018.03059 30619378 PMC6306491

[37] Sharma S, Zhu L, Srivastava MK, Harris-White M, Huang M, Lee JM, Rosen F, Lee G, Wang G, Kickhoefer V, Rome LH, Baratelli F, St John M, Reckamp K, Chul-Yang S, Hillinger S, Strieter R, Dubinett S. 2013. CCL21 chemokine therapy for lung cancer. Int Trends Immun 1:10–15. doi:10.1007/978-1-4614-6613-0_33-4 25264541 PMC4175527

[38] Conklin L, Hviid A, Orenstein WA, Pollard AJ, Wharton M, Zuber P. 2021. Vaccine safety issues at the turn of the 21st century. BMJ Glob Health 6:e004898. doi:10.1136/bmjgh-2020-004898 PMC8137241 34011504

[39] Ali RH, Ali ME, Samir R. 2022. Production and characterization of bacterial ghost vaccine against Neisseria meningitidis. Vaccines (Basel) 11:37. doi:10.3390/vaccines11010037 36679882 PMC9865227

[40] Halder P, Maiti S, Banerjee S, Das S, Dutta M, Dutta S, Koley H. 2023. Bacterial ghost cell based bivalent candidate vaccine against Salmonella Typhi and Salmonella Paratyphi A: a prophylactic study in BALB/c mice. Vaccine (Auckl) 41:5994–6007. doi:10.1016/j.vaccine.2023.08.049 37625993

[41] Mirali M, Jahangiri A, Jalali Nadoushan M, Rasooli I. 2023. A two-protein cocktail elicits a protective immune response against Acinetobacter baumannii in a murine infection model. Microb Pathog 182:106262. doi:10.1016/j.micpath.2023.106262 37474079

[42] Islam MM, Miura S, Hasan MN, Rahman N, Kuroda Y. 2020 Anti-dengue ED3 long-term immune response with T-cell memory generated using solubility controlling peptide tags. Front Immunol 11:333. doi:10.3389/fimmu.2020.00333 32256488 PMC7089932

[43] Kawabe T, Ciucci T, Kim KS, Tayama S, Kawajiri A, Suzuki T, Tanaka R, Ishii N, Jankovic D, Zhu J, Sprent J, Bosselut R, Sher A. 2022. Redefining the foreign antigen and self-driven memory CD4^+^ T-cell compartments via transcriptomic, phenotypic, and functional analyses. Front Immunol 13:870542. doi:10.3389/fimmu.2022.870542 35707543 PMC9190281

[44] Lanzavecchia A, Sallusto F. 2005. Understanding the generation and function of memory T cell subsets. Curr Opin Immunol 17:326–332. doi:10.1016/j.coi.2005.04.010 15886125

[45] Jin L, Jin L, Wu R, Liu X, Zhu X, Shou Q, Fu H. 2020. Hirsutella sinensis fungus regulates CD8^+^ T cell exhaustion through involvement of T-Bet/Eomes in the tumor microenvironment. Front Pharmacol 11:612620. doi:10.3389/fphar.2020.612620 33488388 PMC7820905

[46] Gebhardt T, Wakim LM, Eidsmo L, Reading PC, Heath WR, Carbone FR. 2009. Memory T cells in nonlymphoid tissue that provide enhanced local immunity during infection with herpes simplex virus. Nat Immunol 10:524–530. doi:10.1038/ni.1718 19305395

[47] Nazeri S, Zakeri S, Mehrizi AA, Sardari S, Djadid ND. 2020. Measuring of IgG2c isotype instead of IgG2a in immunized C57BL/6 mice with Plasmodium vivax TRAP as a subunit vaccine candidate in order to correct interpretation of Th1 versus Th2 immune response. Exp Parasitol 216:107944. doi:10.1016/j.exppara.2020.107944 32619431

[48] Khan IU, Ahmad F, Zhang S, Lu P, Wang J, Xie J, Zhu N. 2019. Respiratory syncytial virus F and G protein core fragments fused to HBsAg-binding protein (SBP) induce a Th1-dominant immune response without vaccine-enhanced disease. Int Immunol 31:199–209. doi:10.1093/intimm/dxy078 30462215

[49] Michalek J, Hezova R, Turanek-Knötigova P, Gabkova J, Strioga M, Lubitz W, Kudela P. 2017. Oncolysate-loaded Escherichia coli bacterial ghosts enhance the stimulatory capacity of human dendritic cells. Cancer Immunol Immunother 66:149–159. doi:10.1007/s00262-016-1932-4 27864613 PMC11029152

[50] Fitzgerald KA, Kagan JC. 2020. Toll-like receptors and the control of immunity. Cell 180:1044–1066. doi:10.1016/j.cell.2020.02.041 32164908 PMC9358771

[51] Xia P, Wu Y, Lian S, Yan L, Meng X, Duan Q, Zhu G. 2021. Research progress on Toll-like receptor signal transduction and its roles in antimicrobial immune responses. Appl Microbiol Biotechnol 105:5341–5355. doi:10.1007/s00253-021-11406-8 34180006 PMC8236385

[52] McAleer JP, Vella AT. 2010. Educating CD4 T cells with vaccine adjuvants: lessons from lipopolysaccharide. Trends Immunol 31:429–435. doi:10.1016/j.it.2010.08.005 20880743 PMC2967613

[53] Muhammad A, Champeimont J, Mayr UB, Lubitz W, Kudela P. 2012. Bacterial ghosts as carriers of protein subunit and DNA-encoded antigens for vaccine applications. Expert Rev Vaccines 11:97–116. doi:10.1586/erv.11.149 22149712

[54] Felnerova D, Kudela P, Bizik J, Haslberger A, Hensel A, Saalmuller A, Lubitz W. 2004. T cell-specific immune response induced by bacterial ghosts. Med Sci Monit 10:BR362–BR370. 15448589

[55] Arts RJW, Moorlag SJCFM, Novakovic B, Li Y, Wang S-Y, Oosting M, Kumar V, Xavier RJ, Wijmenga C, Joosten LAB, Reusken CBEM, Benn CS, Aaby P, Koopmans MP, Stunnenberg HG, van Crevel R, Netea MG. 2018. BCG vaccination protects against experimental viral infection in humans through the induction of cytokines associated with trained immunity. Cell Host Microbe 23:89–100. doi:10.1016/j.chom.2017.12.010 29324233

[56] Gu H, Zeng X, Peng L, Xiang C, Zhou Y, Zhang X, Zhang J, Wang N, Guo G, Li Y, Liu K, Gu J, Zeng H, Zhuang Y, Li H, Zhang J, Zhang W, Zou Q, Shi Y. 2021. Vaccination induces rapid protection against bacterial pneumonia via training alveolar macrophage in mice. eLife 10:e69951. doi:10.7554/eLife.69951 34544549 PMC8455131

